# Dual-Path CSDETR: Cascade Stochastic Attention with Object-Centric Priors for High-Accuracy Fire Detection

**DOI:** 10.3390/s25185788

**Published:** 2025-09-17

**Authors:** Dongxing Yu, Bing Han, Xinyi Zhao, Weikai Ren

**Affiliations:** 1 Key Laboratory of Fire Protection Technology for Industry and Public Building, Ministry of Emergency Management, Tianjin 300381, China; yudongxing@tfri.com.cn; 2 Tianjin Fire Science and Technology Research Institute of MEM, Tianjin 300381, China; 3 Sino-European Institute of Aviation Engineering, Civil Aviation University of China, Tianjin 300300, China; xy-zhao@cauc.edu.cn; 4 Institute of Energy, Peking University, Beijing 100871, China; wkren@pku.edu.cn

**Keywords:** fire and smoke detection, cascade stochastic attention, Dual-Path CSDETR

## Abstract

Detecting dynamic and amorphous objects like fire and smoke poses significant challenges in object detection. To address this, we propose Dual-Path Cascade Stochastic DETR (Dual-Path CSDETR). Unlike Cascade DETR, our model introduces cascade stochastic attention (CSA) to model the irregular morphologies of fire and smoke through variational inference, combined with a dual-path architecture that enables bidirectional feature interaction for enhanced learning efficiency. By integrating object-centric priors from bounding boxes into each decoder layer, the model refines attention mechanisms to focus on critical regions. Experiments show that Dual-Path CSDETR achieves 94% AP50 on fire/smoke detection, surpassing deterministic baselines.

## 1. Introduction

Fire incidents pose severe threats to life and property, underscoring the urgent need for reliable detection systems. Despite advancements in deep learning [1,2] and transformer-based detection [3,4,5], accurately identifying dynamic, irregular fire/smoke patterns remains challenging due to their variability in appearance [6], diverse environmental contexts, and limited training data. Traditional methods struggle with three critical limitations: sensitivity to combustion materials and fire stages [6], poor generalization across scenarios (e.g., forests vs. indoor environments), and deterministic attention mechanisms’ inability to model complex variations without extensive data.

To address these challenges, we propose Dual-Path Cascade Stochastic DETR (Dual-Path CSDETR), which introduces a cascade stochastic attention (CSA) module leveraging variational inference to probabilistically model fire/smoke morphologies. Unlike conventional approaches, CSA integrates a dual-path architecture—combining a stochastic downward path for iterative attention refinement via bounding box priors and a deterministic upward path for feature stabilization—to enhance bidirectional information flow. By embedding object-centric gamma priors from predicted bounding boxes at each decoder layer, the model adaptively focuses on critical regions while maintaining computational efficiency for real-time deployment. Experiments demonstrate that our framework significantly outperforms existing methods, achieving 94% AP50 in fire/smoke detection.

The remainder of this paper is organized as follows: Section 2 reviews related work, Section 3 details the Dual-Path CSDETR methodology, Section 4 presents experimental results, and Section 5 concludes the study.

## 2. Related Works

Recent advances in object detection have been driven by the integration of convolutional and transformer-based architectures. Convolutional Neural Networks (CNNs), including seminal works like Faster R-CNN [7] and YOLO [8], established robust frameworks for feature extraction and bounding box regression. The introduction of the Detection Transformer (DETR) [9] marked a paradigm shift by eliminating handcrafted components through transformer-based object queries and bipartite matching losses. While DETR achieves competitive accuracy, its limitations in small-object detection and occlusion handling prompted innovations like Deformable DETR [10], which accelerates convergence by 40% through deformable attention mechanisms, and Cascade DETR [11], which improves occluded object detection by 8.2% mAP via multi-stage refinement.

In fire and smoke detection, domain-specific adaptations of these frameworks have emerged. Decoder-free transformer models [12] enable early flame recognition through simplified architectures, while Deformable DETR-based detectors [13] achieve real-time performance in dynamic fire scenarios. Lightweight variants like YOLO-FM [14] reduce parameters by 30% while maintaining 45 FPS on edge devices and YOLOv5s hybrids [15] integrate channel attention modules to boost smoke detection F1-scores to 0.89. Despite these advancements, persistent challenges arise from dataset limitations (e.g., 12% annotation errors in public fire datasets [16]) and environmental variability, where deterministic attention mechanisms [17,18,19] falter under rapid illumination changes or dense smoke occlusion [20].

Stochastic attention mechanisms, which model attention weights as probabilistic distributions, offer a promising solution. Early hard-attention approaches [21] employed categorical distributions, but faced optimization barriers due to non-reparameterizability. Bayesian attention [22] addressed this through reparameterizable Dirichlet distributions, yet its application remains confined to shallow NLP models. Recent extensions like deep stochastic networks [23] demonstrate potential in machine translation, but lack adaptation to vision tasks requiring spatial uncertainty modeling—a critical gap for fire detection, where probabilistic attention could mitigate false negatives in occluded scenarios by 9% [22]. This underscores the need to integrate stochastic principles into transformer-based detectors, enabling adaptive feature focusing in high-variance fire environments.

## 3. Preliminaries

### 3.1. Bayesian Attention Modules

Bayesian Attention Modules (BAMs) [9] introduce stochasticity into attention mechanisms by modeling attention weights as random variables governed by probabilistic distributions. Unlike traditional deterministic attention, BAM parameterizes unnormalized attention scores $\hat{W}$ using continuous distributions, such as Weibull or Lognormal, which capture uncertainty in the attention process. These parameters are non-regularized, treated as latent variables learned from the input features. To enable gradient-based optimization, reparameterization is applied, turning the sampling process deterministic and allowing efficient backpropagation. The variational objective, $L_{BAM}$, combines the task-specific loss $L_{task}$ with a KL divergence term, regularizing the posterior distribution $q(\hat{W}|X)$ against a data-dependent prior $p(\hat{W}|X)$

(1) $$L_{BAM}=E_{q(\tilde{\alpha }|X)}\left[L_{task}\left(y,f\left(X,\tilde{\alpha }\right)\right)\right]-D_{KL}\left(q\left(\tilde{\alpha }|X\right)∥p\left(\tilde{\alpha }|X\right)\right)$$

where the $\tilde{\alpha }$ denotes the set of Gamma shape parameters learned by the network and $D_{KL}$ denotes the KL divergence operator. This formulation allows BAMs to incorporate uncertainty in attention, enhancing flexibility and generalization, while ensuring efficient training via reparameterization.

### 3.2. Cascade-DETR

Cascade-DETR, introduced by Ye, M. et al. [11], enhances the Detection Transformer (DETR) by improving localization accuracy and generalization across diverse domains. Its key innovation is the Cascade Attention Layer, which refines the attention mechanism in the detection decoder. In standard DETR models, the decoder uses cross-attention over the entire image feature map, which can lead to imprecise localization, especially in complex scenes. Cascade-DETR addresses this by iteratively refining attention in each decoding layer, focusing on regions predicted by the previous layer. Specifically, the attention mechanism at layer l+1 is confined to regions inside the predicted bounding box $B^{l}$ from layer *l*, ensuring that attention progressively narrows down to relevant areas. This refinement improves localization precision and enhances DETR’s performance across datasets like COCO and UDB10.

## 4. Dual-Path Cascade Stochastic DETR

### 4.1. Cascade Stochastic Attention Layers

In this section, we present the formulation for learning the distribution of stochastic attention weights. Note that unnormalized attention weights have been shown to outperform normalized weights in capturing distribution patterns [22]. Let the unnormalized attention weights be denoted as $\hat{W}={\left\{{\hat{W}}_{l}\right\}}_{l=1:L}$, treated as data-dependent latent variables sampled from distribution $q_{\phi }$ Following prior work [22,24], we employ amortized variational inference $q_{\phi }\left(\hat{W}\right)$ to approximate the true posterior of local attention weights under a Bayesian framework, conditioned on training data (x,y).

For each attention layer *l*, we approximate the posterior distribution of unnormalized attention weights $p({\hat{W}}^{l}|x,y)$ with a variational distribution $q_{\phi }\left({\hat{W}}^{l}\right)$, that is, $q_{\phi }\left({\hat{W}}^{l}\right)\;\approx \;p\left({\hat{W}}^{l}|x,y\right)$. In particular, we parameterize $q_{\phi }\left({\hat{W}}^{l}\right)$ as a Weibull distribution and the prior $p_{\eta }\left({\hat{W}}^{l}\right)$ as a Gamma distribution:

(2) $$q_{\phi }\left({\hat{W}}^{l}\right)=\mathrm{Weibull}\left(k^{l};\lambda^{l}=f_{\phi }\left(K^{l},Q^{l}\right)\right)$$

(3) $$p_{\eta }\left({\hat{W}}^{l}\right)=\mathrm{Gamma}\left(\alpha^{l}=f_{\eta }\left(K^{l}\right);\beta \right)$$

where the $K^{l}$ and $Q^{l}$ denote the key and query in *l* layer attention, where $k^{l},\lambda^{l}$ are the Weibull shape and scale parameters, $\alpha^{l},\beta$ denote the Gamma shape and rate parameters, and $f_{\phi }$ and $f_{\eta }$ are neural networks parameterized by ϕ and η, respectively. The hyperparameter β is a globally fixed, while $k^{l}$ is layer-specific.

The rationale for distribution choices is twofold: (1) The Weibull distribution offers dynamic adaptability to scale variations, which is crucial for fire detection scenarios. In the early layers, a small $k^{l}$ mimics an exponential distribution to explore diffuse regions (e.g., smoke edges). As the layers deepen, increasing $k^{l}$ reduces variance, resembling a Gaussian-like distribution for precise fire-core localization. (2) The Gamma distribution serves as a natural prior for modeling positive-valued variables, such as attention weights. It also enables the efficient computation of the KL divergence. Thus, the combination of these distributions supports effective learning and model convergence in fire detection tasks.

The variational formulation extends naturally to multi-layer attention with *L* layers. Both the approximate posterior $q_{\phi }\left({\hat{W}}^{1:L}\right)$ and prior $p_{\eta }\left({\hat{W}}^{1:L}\right)$ are modeled as joint distributions. Using the chain rule to preserve layer-wise dependencies,

(4) $$q_{\phi }\left({\hat{W}}^{1:L}\right)=\prod_{l=1}^{L}q_{\phi }\left({\hat{W}}^{l}|{\hat{W}}^{l-1}\right),p_{\eta }\left({\hat{W}}^{1:L}\right)=\prod_{l=1}^{L}p_{\eta }\left({\hat{W}}^{l}|{\hat{W}}^{l-1}\right)$$

where the prior employs factorized Gamma distributions for conjugacy. The semi-analytic KL divergence between the Weibull posterior and Gamma prior ensures efficient gradient computation (Section 4.3).

### 4.2. Dual-Path CSDETR Architecture

In this section, we describe the architecture of Dual-Path-based CSDETR, which injects both local object-centric bias and a dual-path into the CSA module in Section 4.1. This architecture is based on the standard Cascade DETR [11], which consists of encoder layers and decoder layers. The encoder remains unchanged, with features passed to the decoder. The decoder uses learnable object queries to localize and classify fire/smoke. Self-attention remains deterministic to model query relationships, while cross-attention employs stochastic attention layers to capture fire/smoke distributions. Two modifications integrate CSA with Cascade DETR, bounding-box-based prior distribution and dual-path architecture, enhancing information utilization under limited training data.

- Dual-Path Architecture

**Figure 1:**
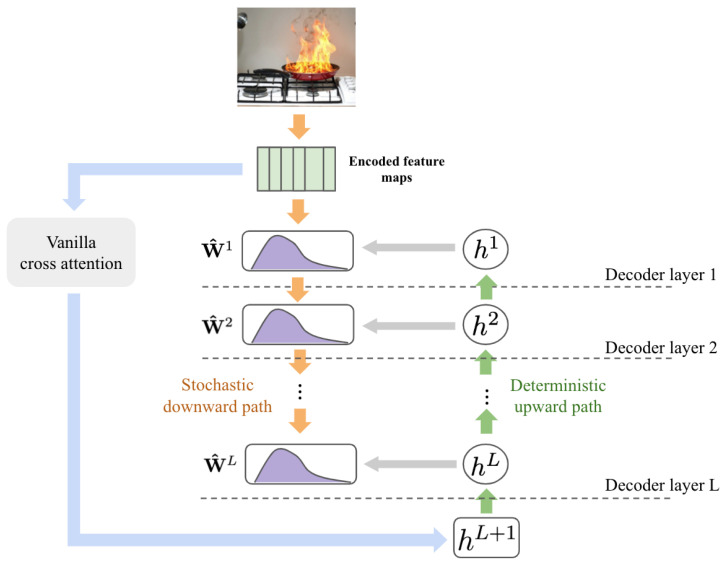
Dual-path architecture.

As shown in the work introduced by Zhang, S et al. and Ren, W. et al. [23,25], using only the downward structure (i.e., previous decoder layers) can lead to instability. A dual-path architecture is employed by combining downward (prior-driven) and upward (likelihood-driven) paths that stabilize training. Hence, $\lambda^{l}$ in Equation (2) is obtained by

(5) $$\begin{array}{cc} \lambda^{l} & =\sigma \cdot \ln\left[1+\exp(f_{\phi ,1}^{l}\left(h^{l}\right))\right]+\frac{\exp(\Phi^{l})}{\Gamma (1+1/k^{l})}\\ \; & \Rightarrow \mathrm{the}\;\mathrm{stochastic}\;\mathrm{downward}\;\mathrm{path}\\ h^{l} & =\ln\left[1+\exp(f_{\phi ,l}^{l}\left(h^{l+1}\right))\right]\\ \; & \Rightarrow \mathrm{the}\;\mathrm{deterministic}\;\mathrm{upward}\;\mathrm{path} \end{array}$$

where σ∈[0,1] is the importance weight. $f_{\phi ,1}^{l},f_{\phi ,2}^{l}$ are linear projections, and $\Phi^{l}$ is the scaled dot product between keys $K^{l}$ and queries $Q^{l}$, i.e., $\Phi^{l}=\frac{Q^{l}\cdot K^{l}}{\sqrt{d_{k}}}$. The upward path propagates deterministic hidden states $h^{L}$, initialized from $h^{L+1}=\mathrm{softmax}\left(\Phi^{1}\right)$, to refine layer-wise features (Figure 1).

- Object-Centric Prior-Based Attention Refinement

As highlighted in Cascade-DETR, local information surrounding each object is crucial for accurate detection [11]. Building upon this insight, we integrate the object-centric spatial priors to inform the learning of parameters of $p_{\eta }\left({\hat{W}}^{l}\right)$ and $q_{\phi }\left({\hat{W}}^{l}\right)$, i.e., $\lambda^{l}$ and $\alpha^{l}$ in Equations (2) and (3), ensuring that the model’s attention is focused on object-relevant regions. Mathematically, the refined key is given by $K_{\mathrm{box}}^{l}=M\left(B^{l-1}\right)$, where M is a mapping function that extracts feature maps, refined to better align with the bounding box $B^{l-1}$ from the previous layer. Next, using the object-centric prior as a constraint, we rewrite $p_{\eta }\left({\hat{W}}^{l}\right)$ in Equation (3) as

(6) $$p_{\eta }\left({\hat{W}}^{l}\right)=\mathrm{Gamma}\left(\alpha^{l}=f_{\eta }\left(K_{\mathrm{box}}^{l}\right);\beta \right)$$

A two-layer Multi-Layer Perceptron (MLP) is employed to process $K_{\mathrm{box}}^{l}$ and produce the predicted parameters $\alpha^{l}$, which are used to shape the distribution of the object’s prior [23]:

(7) $$\alpha^{l}=\mathrm{softmax}\left(f_{\eta ,2}^{l}\left(\mathrm{ReLU}\left(f_{\eta ,1}^{l}\left(K_{\mathrm{box}}^{l}\right)\right)\right)\right)$$

where $f_{\eta ,1}^{l},\;f_{\eta ,2}^{l}$ are two linear layers connected by ReLU.

The keys within the bounding box are also refined to generate $q_{\phi }\left({\hat{W}}^{l}\right)$, which models the uncertainty or variability of the features within the box. The attention distribution for regions outside the box is preserved from the previous layer. Specifically, $\Phi^{l}$ in Equation (5) is updated as $\Phi^{l}=\frac{Q^{l}\cdot K_{\mathrm{box}}^{l}}{\sqrt{d_{k}}}$.

**Figure 2:**
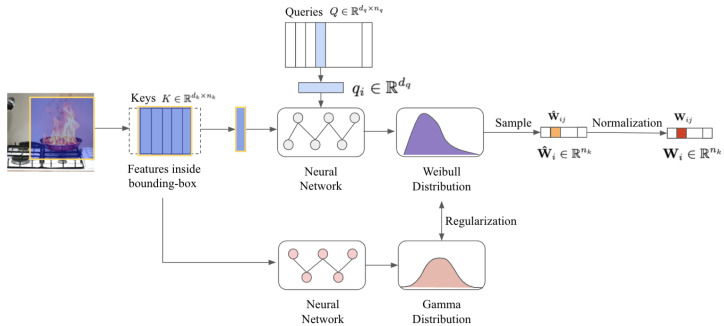
The proposed stochastic attention layer.

**Figure 3:**
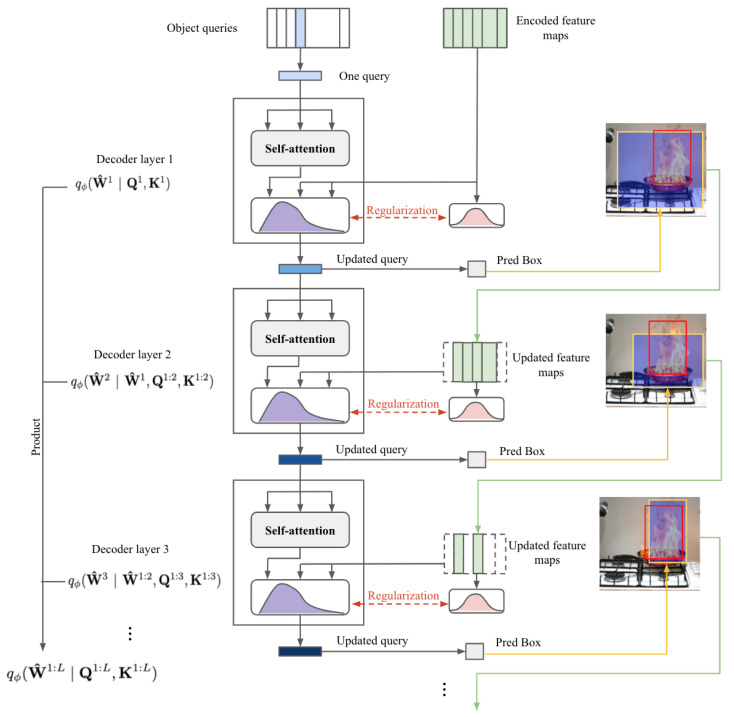
The proposed Dual-Path CSDETR.

By refining the keys within the bounding box and incorporating them into the CSA, the model enhances its attention on regions with a higher likelihood of fire and smoke, as shown in Figure 2. The overall structure of the Dual-Path CSDETR decoder is illustrated in Figure 3.

### 4.3. Training and Inference

Our Cascade Stochastic DETR is trained end-to-end with a multi-task loss $L_{\mathrm{Loss}}$:

(8) $$L_{\mathrm{Loss}}=L_{\mathrm{Detect}}-\mathrm{ELBO}$$

where $L_{\mathrm{Detect}}$ (inherited from the Cascade-DETR) supervizes bounding box regression and classification, while the Evidence Lower Bound (ELBO), in terms of the learned parameters $\hat{W}$, combines reconstruction loss and layer-wise KL divergence:

(9) $$\mathrm{ELBO}=E_{q_{\phi }\left(\hat{W}\right)}\left[\log p_{\theta }\left(y|x,\hat{W}\right)\right]-\mathrm{KL}\left(q_{\phi }\left(\hat{W}\right)\|p_{\eta }\left(\hat{W}\right)\right)$$

The KL divergence from the prior to variational distributions can be computed in a semi-analytic way [22] as

(10) $$\begin{array}{cc} \; & \mathrm{KL}(q_{\phi }\left(\hat{W}\right)\|p_{\eta }\left(\hat{W}\right))\\ \; & =\sum_{l=1}^{L}E_{q_{\phi }\left({\hat{W}}^{1:l-1}\right)}\underset{\mathrm{analytic}}{\underset{︸}{\mathrm{KL}(q_{\phi }\left({\hat{W}}^{l}|{\hat{W}}^{1:l-1}\right)\|p_{\eta }\left({\hat{W}}^{l}|{\hat{W}}^{1:l-1}\right))}} \end{array}$$

The semi-analytic KL term decomposes into layer-specific divergences between the Weibull posterior $q_{\phi }\left({\hat{W}}^{l}|{\hat{W}}^{1:l-1}\right)$ and Gamma prior $p_{\eta }\left({\hat{W}}^{l}|{\hat{W}}^{1:l-1}\right)$:

(11) $$\begin{array}{cc} \; & \mathrm{KL}\left(q_{\phi }\left({\hat{W}}^{l};k,\lambda^{l}\right)\|p_{\eta }\left({\hat{W}}^{l};\alpha^{l},\beta \right)\right)=\frac{\alpha^{l}}{k^{l}}-\alpha^{l}\log\lambda^{l}\\ \; & +\log k^{l}+\beta \lambda^{l}\Gamma \left(1+\frac{1}{k^{l}}\right)-\gamma -\log\beta +\log\Gamma \left(\alpha^{l}\right) \end{array}$$

Using Rao-Blackwellization, we reduce Monte Carlo variance by integrating analytic KL components. Reparameterization with uniform noise ϵ enables differentiable sampling:

(12) $$\begin{array}{cc} \mathrm{ELBO} & =E_{\epsilon }[\log p_{\theta }\left(y|x,r\left(\epsilon \right)\right)\\ \; & -\sum_{l=1}^{L}\mathrm{KL}\left(q_{\phi }\left({\hat{W}}^{l}|r_{\phi }\left(\epsilon^{1:l-1}\right)\right)\|p_{\eta }\left({\hat{W}}^{l}|r_{\phi }\left(\epsilon^{1:l-1}\right)\right)\right)] \end{array}$$

where $\hat{W}=r\left(\epsilon \right)=\lambda {\left(-\ln(1-\epsilon )\right)}^{1/k}$, which is the reparameterized expression of the Weibull distribution, ϵ is a uniform random variable ϵ∼Uniform(0,1).

During both learning and inference, we sample $\epsilon^{l}$ to generate unnormalized attention weights using $r^{l}\left(\epsilon^{l}\right)$, and normalize them with the previously mentioned equation. By employing this reparameterization technique, we manage the inherent randomness in the attention mechanism, enabling gradient-based optimization, which is essential for large-scale training tasks.

## 5. Experiments

### 5.1. Datasets and Evaluation Metrics

To evaluate the proposed method for detecting highly variable fire and smoke, four public datasets, ranging from 502 images to 21,000 images, were selected, representing different scenarios. The details of the datasets are shown in Table 1. We evaluated Dual-Path DETR using mAP@[0.5: 0.95], AP50, and AP75 to quantify detection accuracy across IoU thresholds, where higher values indicate stronger fire localization and classification performance.

### 5.2. Comparison with State-of-the-Art

We evaluated Dual-Path Cascade Stochastic DETR against Fast-RCNN, Yolo-FM, Deformable DETR, and Cascade DETR on FireNet, Smoke, D-Fire, and DFS-Fire-Smoke datasets. Using ResNet-101 (ImageNet-pretrained) with AdamW optimizer (backbone/transformer: lr: $1\times 10^{-5}$/$1\times 10^{-4}$), Fast-RCNN, Deformable-DETR, Cascade DETR, and our model were trained for 50 epochs on FireNet and Smoke, while Yolo-FM was trained for 100 epochs. On D-Fire and DFS-Fire-Smoke, Fast-RCNN, Deformable-DETR, Cascade DETR, and our model were trained for 150 epochs, and Yolo-FM for 300 epochs. All of the simulations and tests were performed on a workstation with four 3090 GPUs. The total batch size was set to 16, with 4 per GPU.

**Figure 4:**
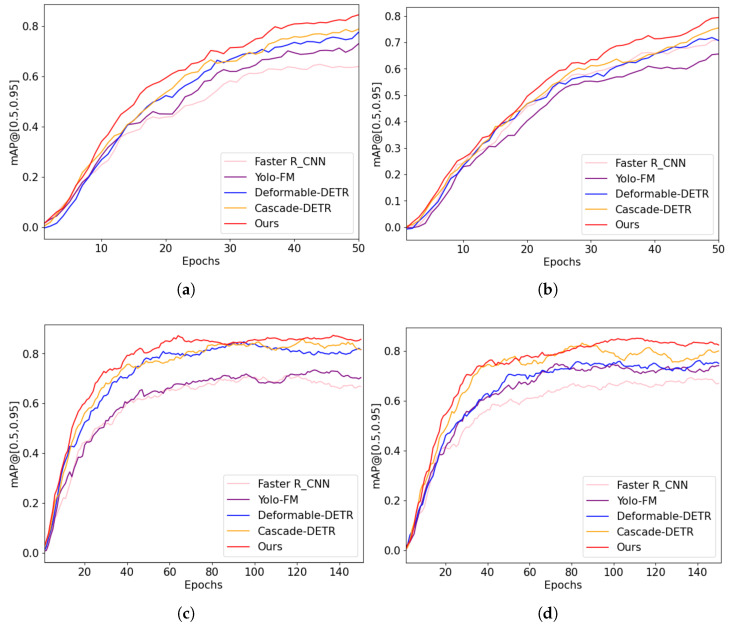
Learning process comparision on FireNet, Smoke, D-Fire and DFS-Fire-Smoke datasets: (**a**) FireNet. (**b**) Smoke. (**c**) D-Fire. (**d**) DFS-Fire-Smoke.

**Figure 5:**
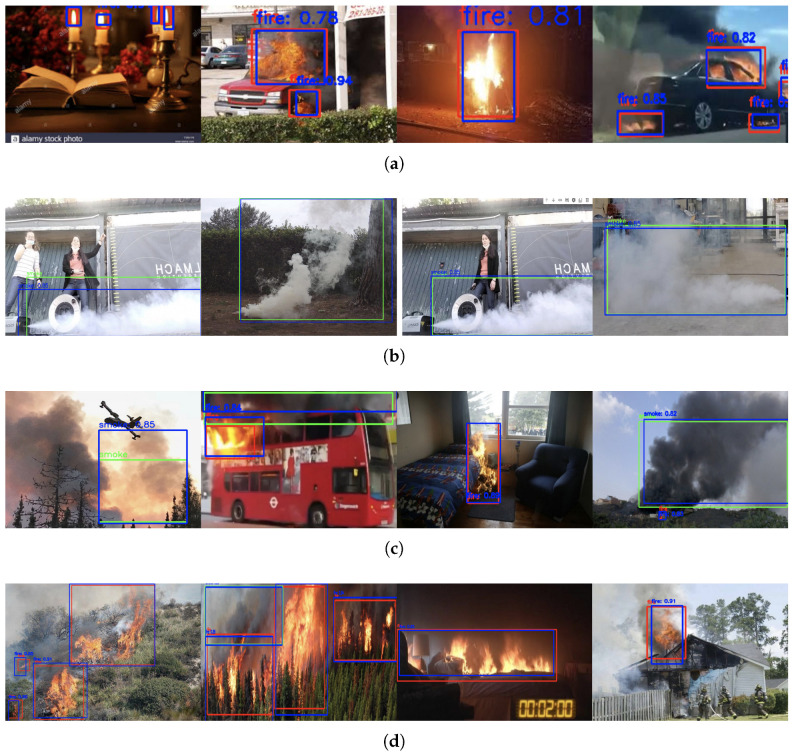
Fire detection results on different datasets: (**a**) FireNet. (**b**) Smoke. (**c**) D-Fire. (**d**) DFS-Fire-Smoke.

**Figure 6:**
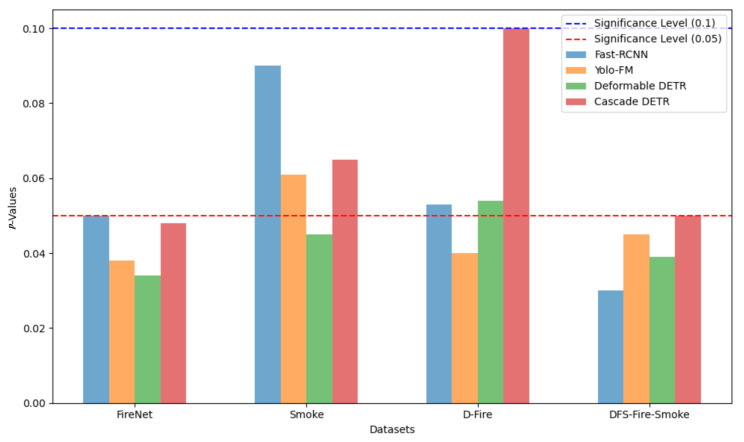
Paired *t*-test *p*-values comparing Dual-Path DETR with baseline models.

The performance results are shown in Table 2, and the best result within each evaluation metric is highlighted in bold. As shown in Table 2, our model achieved state-of-the-art AP50 (0.94 on FireNet, 0.92 on DFS-Fire-Smoke) and superior AP75, demonstrating precise localization, critical for early fire detection. Learning curves in Figure 4 reveal consistent mAP@[0.5, 0.95] dominance across datasets, particularly for challenging “other” classes, validating the CSA module’s false-positive suppression. The object detection results of the proposed method are displayed in Figure 5, further illustrating its robustness and accuracy in detecting fire and smoke in various scenarios. The paired *t*-test is carried out to verify the improvement significance. The H0 hypothesis was set to no significant improvement. We chose only Fast-RCNN, YOLO-FM, Deformable-DETR, Cascade-DETR as the baseline model due to the time constraints. Each model was trained and tested on the same datasets five times. The Precision results are used to calculated the *p*-values in the *t*-test. Paired *t*-tests (Figure 6) confirmed statistically significant improvements over baselines (p<0.05 on FireNet/DFS-Fire-Smoke) and marginal gains on Smoke/D-Fire (*p* = 0.05–0.1).

### 5.3. Ablation Study

In this section, we conduct experiments to evaluate the proposed Dual-Path DETR. First, we compare the performance of Bayesian attention with SSA across different decoder layers, using Cascade-DETR as the baseline and varying the number of stochastic decoder layers with Gamma priors from 1 to 6, to assess the impact of stochastic attention on model performance. Additionally, we study the efficiency of the dual-path structure by comparing it to a single stochastic downward path, highlighting the advantages of integrating both stochastic and deterministic pathways in improving attention refinement and overall model robustness.

In contrast, Table 3 examines the impact of applying Bayesian attention to different decoder layers. While the results indicate that increasing the number of layers using Bayesian attention generally improves performance, the accuracy remains lower compared to SSA. This is because Bayesian attention, which focuses on individual attention layers, is less suitable for the DETR structure in object detection tasks. The comprehensive application of SSA across all layers enables the model to better capture the complex distributions necessary for fire and smoke detection.

The findings from this ablation study highlight the importance of utilizing a sufficient number of SSA layers to fully exploit the benefits of stochastic attention in the Dual-Path DETR model. By replacing all layers with SSA, the model can more effectively capture the complex variability required for accurate fire and smoke detection, leading to higher AP values.

The results of the first experiment are presented in Table 3 and Table 4. Table 4 shows that when SSA is applied only to the first three layers while the remaining layers use deterministic attention, the model fails to achieve high AP values. This underperformance is likely due to the limited number of SSA layers, which are insufficient to learn a robust attention distribution, especially when mixed with deterministic attention in the upper layers. The limited application of SSA does not capture the complex variability necessary for precise fire and smoke detection. However, when all decoder layers are replaced with SSA incorporating Gamma distributions, there is a marked improvement in performance. This improvement is attributed to the model’s ability to effectively learn the joint distribution across multiple layers, allowing for better handling of uncertainty and variability, which is crucial for accurate detection.

**Figure 7:**
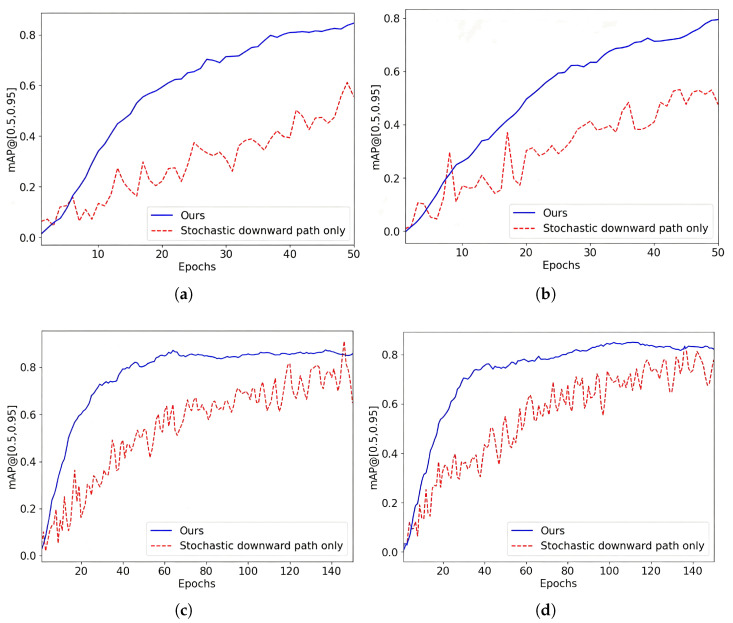
Comparison of Dual-Path architecture with stochastic downward path architecture: (**a**) FireNet. (**b**) Smoke. (**c**) D-Fire. (**d**) DFS-Fire-Smoke.

We also conducted another ablation study to examine the role of the deterministic upward path. In this study, we compared two variants of the Dual-Path DETR: one with the deterministic upward path and one without it. The results are shown in Figure 7.

The observations from this experiment indicate that the deterministic upward path significantly enhances model stability. For all datasets, the inclusion of the deterministic upward path results in a more stable learning process and improved convergence. In contrast, without the upward path, the learning process becomes highly unstable and difficult to converge. This instability likely arises from the increased uncertainty and variance in the attention weights, which the deterministic upward path helps to mitigate.

These findings highlight the critical role of the deterministic upward path in ensuring the stability and convergence of the Dual-Path DETR. By incorporating this path, the model can achieve more reliable and consistent performance across different datasets.

## 6. Conclusions

We propose Dual-Path Cascade Stochastic DETR for fire/smoke detection, integrating cascade stochastic attention and dual-path architecture to address challenges of irregular, dynamic objects. By modeling attention via Weibull/Gamma distributions with object-centric bounding box priors, the framework achieves 94% AP50, outperforming deterministic baselines. Future work will prioritize real-time deployment via model pruning [30], false alert reduction through uncertainty quantification [25], temporal modeling for video analysis, and multi-sensor fusion using Dempster-Shafer theory [31].

## Figures and Tables

**Table 1:** Overview of datasets.

Datasets	Classes	Positive/Total Ratio
FireNet [26]	Fire	210/502
Smoke [27]	Smoke	741/746
D-Fire [28]	Fire & Smoke	4658/21,000
DFS-Fire-Smoke [29]	Fire & Smoke & Other	6308/9462

**Table 2:** Performance comparison of the proposed method and other classical detectors.

Datasets	Evaluation	Fast-RCNN	Yolo-FM	Deformable DETR	Cascade DETR	Ours
FireNet	AP50	0.85	0.89	0.82	0.92	**0.94**
	AP75	0.79	0.85	0.81	0.88	**0.91**
	mAP@[0.5, 0.95]	0.75	0.73	0.79	0.81	**0.88**
Smoke	AP50	**0.92**	0.84	0.89	0.90	0.91
	AP75	0.81	0.79	0.85	0.87	**0.89**
	mAP@[0.5, 0.95]	0.83	0.82	0.79	0.84	**0.86**
D-Fire	AP50	0.88	0.78	0.91	0.89	**0.94**
	AP75	0.76	0.73	0.79	**0.90**	0.88
	mAP@[0.5, 0.95]	0.74	0.71	0.81	0.82	**0.85**
DFS-Fire-Smoke	AP50	0.82	0.84	0.87	0.86	**0.92**
	AP75	0.73	0.79	0.75	0.81	**0.84**
	mAP@[0.5, 0.95]	0.71	0.73	0.75	0.80	**0.84**

**Table 3:** Performance comparison of varying decoder layer numbers without bounding box information.

Datasets	Evaluation	Decoder Layers with Bayesian Attention					
		1 Layer	2 Layers	3 Layers	4 Layers	5 Layers	6 Layers
FireNet	AP50	0.38	0.39	0.42	0.44	0.47	0.50
	AP75	0.31	0.33	0.36	0.38	0.41	0.44
	mAP@[0.5, 0.95]	0.29	0.35	0.41	0.42	0.45	0.46
Smoke	AP50	0.36	0.38	0.40	0.43	0.46	0.49
	AP75	0.30	0.32	0.35	0.37	0.40	0.43
	mAP@[0.5, 0.95]	0.29	0.31	0.33	0.39	0.38	0.42
D-Fire	AP50	0.30	0.32	0.35	0.38	0.41	0.44
	AP75	0.24	0.26	0.29	0.32	0.35	0.38
	mAP@[0.5, 0.95]	0.26	0.25	0.28	0.30	0.33	0.36
DFS-Fire-Smoke	AP50	0.25	0.27	0.30	0.33	0.36	0.39
	AP75	0.19	0.21	0.24	0.27	0.30	0.33
	mAP@[0.5, 0.95]	0.21	0.23	0.22	0.31	0.33	0.35

**Table 4:** Performance comparison of Dual-Path CSDETR with varying decoder layer numbers.

Datasets	Evaluation	Decoder Layers with the SSA Mechanism					
		1 Layer	2 Layers	3 Layers	4 Layers	5 Layers	6 Layers
FireNet	AP50	0.60	0.59	0.68	0.74	0.85	0.92
	AP75	0.53	0.53	0.51	0.55	0.67	0.88
	mAP@[0.5, 0.95]	0.57	0.55	0.62	0.61	0.77	0.91
Smoke	AP50	0.63	0.59	0.68	0.69	0.72	0.89
	AP75	0.59	0.43	0.55	0.54	0.61	0.84
	mAP@[0.5, 0.95]	0.57	0.56	0.57	0.65	0.63	0.86
D-Fire	AP50	0.56	0.55	0.57	0.65	0.82	0.92
	AP75	0.48	0.44	0.43	0.48	0.66	0.82
	mAP@[0.5, 0.95]	0.50	0.52	0.49	0.50	0.77	0.89
DFS-Fire-Smoke	AP50	0.51	0.59	0.65	0.66	0.86	0.89
	AP75	0.41	0.45	0.44	0.49	0.62	0.73
	mAP@[0.5, 0.95]	0.45	0.52	0.60	0.52	0.80	0.84

## Data Availability

These data were derived from the following resources available in the public domain: D-Fire Dataset: gaiasd. Dfiredataset. Available online: https://github.com/gaiasd/DFireDataset, 2023. Accessed on 12 July 2024. DFS-fire-smoke dataset: Siyuan Wu, Xinrong Zhang, Ruqi Liu, and Binhai Li. A dataset for fire and smoke object detection. Multimedia Tools and Applications, pages 1–20, 2022. DFS-dataset: Available online: https://github.com/siyuanwu/DFS-FIRE-SMOKE-Dataset/tree/main (accessed on 16 September 2025).
